# Case report and literature review of pleural myeloid sarcoma

**DOI:** 10.1097/MD.0000000000044388

**Published:** 2025-11-21

**Authors:** Xiaolin Wang, Jiaqiang Liu

**Affiliations:** a Department of Hematology, Rizhao People’s Hospital, Rizhao, Shandong, China.

**Keywords:** case report, diagnosis, myeloid sarcoma, treatment

## Abstract

**Rationale:**

Myeloid sarcoma (MS) is an extramedullary solid tumor composed of myeloid progenitor cells, which is rare in non-leukemic patients. Our research aims to enhance the understanding of the challenges in diagnostic and therapeutic of MS.

**Patient concerns:**

A 61-year-old male was admitted to hospital presenting with “malignant pleural tumor diagnosed 2 months prior, accompanied by chest distress for over a month.”

**Diagnosis:**

Cytological analysis of pleural effusion confirmed malignant cells and supported by immunohistochemical results. Bone marrow biopsy showed 83.5% blasts, with flow cytometry indicating 56.32% tumor cells and the presence of the AML1–ETO fusion gene (FLT3−ITD+). Cytogenetic analysis revealed complex karyotypic abnormalities.

**Interventions and outcomes:**

After treated with the Idarubicin-Cytarabine regimen and intrapleural cisplatin, bone marrow biopsy revealed residual tumor cells (0.46%). Further consolidation with the Idarubicin-Cytarabine regimen and additional cycles of azacitidine plus venetoclax and cytarabine plus venetoclax were administered. Unfortunately, the patient passed away following disease progression.

**Lessons:**

Although pleural myeloid sarcoma is extremely rare, it must be included in the differential diagnosis for unexplained solid pleural masses, particularly when accompanied by pleural effusion. Upon diagnosis, comprehensive staging investigations, including bone marrow biopsy and flow cytometry, must be performed immediately. The successful management of such complex cases relies on the close collaboration of a multidisciplinary team, including radiologists, pathologists, hematologists, and thoracic surgeons. Radiologists identify atypical imaging features, pathologists confirm the diagnosis through precise immunophenotyping, and ultimately, hematologists formulate and execute the correct treatment plan.

## 1. Introduction

Myeloid sarcoma (MS), also termed granulocytic sarcoma or chloroma, is a rare extramedullary neoplasm arising from myeloid precursor cells. It frequently manifests as a progression marker of hematologic malignancies, most commonly acute myeloid leukemia (AML).^[1]^ Isolated myeloid sarcoma (IMS), a distinct subtype, is defined by extramedullary myeloid tumor infiltration without concurrent bone marrow involvement. IMS often progresses to AML or other myelodysplastic disorders.^[2,3]^ MS exhibits a male predominance (male-to-female ratio: 1.2–1.8:1) and can occur at any age, though it is more prevalent in children and young adults.^[4]^ While lymph nodes, skin, and soft tissues are common sites, pleural involvement is exceedingly rare. Diagnostic and therapeutic standardization remains elusive due to the scarcity of cases. Current therapeutic strategies for MS primarily employ AML-based chemotherapy regimens, with hematopoietic stem cell transplantation (HSCT) serving as the definitive treatment modality. The integration of AML chemotherapy protocols and allogeneic hematopoietic stem cell transplantation (allo-HSCT) in non-leukemic MS significantly improves overall survival (OS) and prolongs patient survival. Surgery or radiotherapy alone fails to impede disease progression. Diagnostic and therapeutic standardization remains elusive due to the scarcity of cases. The prognostic value of the Cachexia Index was evaluated for MS.^[5]^ The neutrophil-to-eosinophil ratio incorporation into clinical practice could aid in stratifying patients by risk, thereby facilitating more individualized treatment planning.^[6]^ To date, the attempt to translate in the adjuvant setting the use of agents with a high objective response rate in metastatic disease has not led to noteworthy results.^[7]^ Therefore, the discovery of novel, more effective targeted treatments on the basis of molecular profiling together with the identification of prognostic molecular markers remains an unmet clinical need.^[8]^ For personalized oncology, one of the fundamental points would be to better define genes and pathways involved in tumorigenesis, and provide a novel understanding of the pathophysiological mechanisms underlying the disease.^[9]^ Herein, we report a rare case of pleural MS to underscore its clinical complexity.

## 2. Case presentation

**Figure 1.**
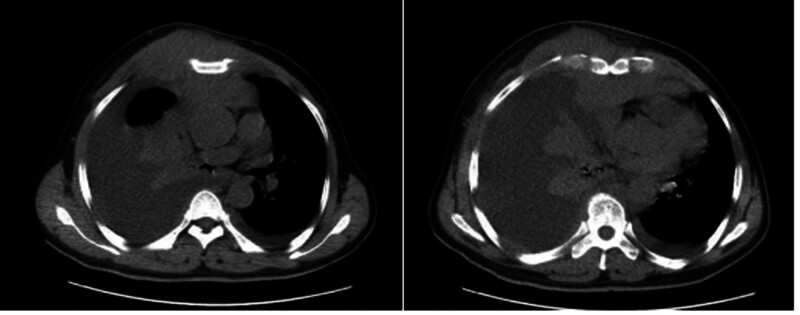
Chest CT images of the patient before treatment. Asymmetric bone density changes are noted in part of the right costal cartilage, and a mass-like soft tissue tumor involving the anterior mediastinum is observed. Bilateral pleural effusion is noted, with massive pleural effusion on the right side associated with right lung atelectasis.

A 61-year-old male was admitted to the Respiratory Medicine Department on November 24, 2022, presenting with “malignant pleural tumor diagnosed 2 months prior, accompanied by chest distress for over a month.” Chest CT revealed heterogeneous ground-glass opacity in the right costal cartilage region, with a soft tissue mass in the anterior mediastinum, pericardial thickening, and bilateral pleural effusion (Fig. 1). Cytological analysis of pleural effusion confirmed malignant cells, supported by immunohistochemical results: CD43+, CD34+, CD79a+, myeloperoxidase focally positive, LCA+, CD117+, CD3−, Ki-67 (~40%), CD68−, TdT−, vimentin−, CK−. The findings suggested myeloid sarcoma, prompting the transfer to the Hematology Department. Bone marrow biopsy showed 83.5% blasts, with flow cytometry indicating 56.32% tumor cells and the presence of the AML1–ETO fusion gene (FLT3−ITD+). Cytogenetic analysis revealed complex karyotypic abnormalities. The complex karyotype is presented as follows: 89<4n>, XX, −Y, −Y, add(1)(q12) −2, −5, der(6)t(1;6)(q21;q23), t(8;21)(q22;q22)x2, −9, −18, +19, +r, +mar.

**Figure 2.**
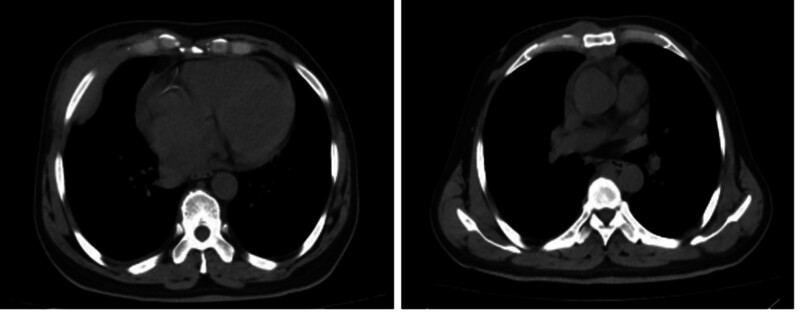
Chest CT images of the patient after receiving consolidation treatment with the IA regimen. A soft tissue nodule in the right thoracic wall, a soft tissue nodule in the posterior mediastinum adjacent to the spine, bilateral pleural thickening, and minimal pleural effusion on the right side.

**Figure 3.**
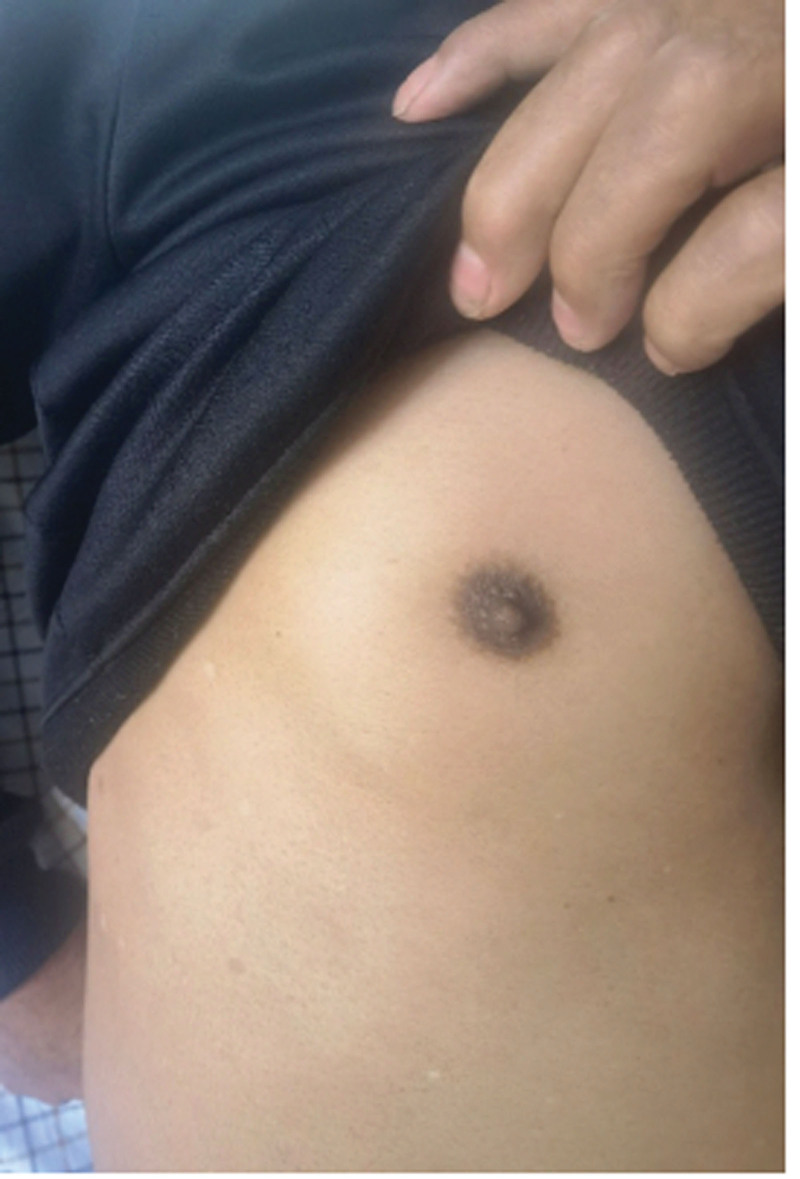
Apparent images of patients. A palpable mass protruding from the right thoracic wall is observed.

Considering disease progression, the patient was treated with the IA regimen and intrapleural cisplatin. Subsequent bone marrow biopsy on February 22, 2023, revealed residual tumor cells (0.46%), with AML1–ETO remaining positive, and the karyotype presented as: 46, XY. The AML1–ETO gene copy number of the patient is 849 with his ABL1 gene copy number is 44,318. The AML1–ETO/ABL ratio of the patient is 1.916% with FLT3–ITD and WT1 turn negative. Bone marrow cytology demonstrates AML–CR (complete remission), but 0.69% abnormal primitive cells were detected in thoracic fluid (Figs. 2 and 3). Further consolidation with the IA regimen and additional cycles of azacitidine plus venetoclax and cytarabine plus venetoclax were administered. Unfortunately, the patient passed away following disease progression.

## 3. Discussion

MS is a rare extramedullary tumor resulting from the proliferation and infiltration of myeloid precursor cells or immature myeloid cells outside the bone marrow. It frequently arises in association with various myeloproliferative disorders but may also present as an initial manifestation. MS can occur across all age groups, predominantly affecting children and young adults, with a higher incidence in males. Clinically, MS often presents as a solitary mass in various tissues or organs or as multiple nodules of varying sizes, with symptoms dependent on the affected anatomical site.

The diagnosis of MS relies on the MICM classification, encompassing morphology, immunology, cytogenetics, and molecular biology. While pathological examination remains the gold standard, sole reliance on morphological assessment can result in a misdiagnosis rate as high as 75%.^[10]^ Common misdiagnoses include diffuse large B-cell lymphoma, Hodgkin lymphoma, T-cell sarcoma, small lymphocytic leukemia, multiple myeloma, and thymoma, with diffuse large B-cell lymphoma being the most prevalent.^[11]^ Immunohistochemistry significantly enhances diagnostic accuracy, reducing the misdiagnosis rate by 25% to 50%.^[12]^ Typical immunophenotypic markers for MS include lysozyme, myeloperoxidase, CD117, CD68, and CD33, which demonstrate high sensitivity but limited specificity.^[13,14]^ Negative expression of CD3 and CD20 helps to exclude T- and B-cell-related tumors.

Recent studies have suggested that interferon regulatory factor 8 may serve as a novel immunological biomarker for MS.^[15]^ When morphology and immunophenotyping are inconclusive, cytogenetic and molecular analyses can provide critical diagnostic information. Approximately 50% of MS cases exhibit chromosomal abnormalities, with chromosome 8 aberrations being the most common. Other frequently observed abnormalities include inv(16), 11q23, t(9;11), t(1;11), deletions in chromosomes 4, 7, 11, 5q−, 16q, and 20.^[16]^ Commonly mutated genes in MS include DNMT3A, FLT3, NPM1, C-Kit, and CEBPA, which are also commonly associated with AML.^[17]^

Imaging techniques play a pivotal role in MS diagnosis, aiding in lesion localization, quantification, and evaluation of treatment response. CT and MRI are highly sensitive and are commonly utilized for detecting suspected MS lesions during early treatment.^[18,19]^ PET/CT, despite its limited specificity, remains useful for identifying extramedullary MS and monitoring therapeutic efficacy.^[12,20]^ The National Comprehensive Cancer Network recommends PET/CT evaluation for AML patients with suspected extramedullary disease.^[21]^ However, due to the brain’s inherently high metabolism, PET/CT is less effective for central nervous system MS, with MRI being preferred for patients exhibiting significant central nervous system-related symptoms.

Fluorodeoxyglucose-PET/CT has demonstrated considerable utility in detecting and localizing metabolic activity in various hematological malignancies.^[22]^ A prospective study reported that 18F-fluorodeoxyglucose-PET/CT possesses high specificity and sensitivity for extramedullary MS, detecting 65 hypermetabolic lesions indicative of MS in 21 of 93 newly diagnosed or relapsed AML adult patients prior to treatment.^[23]^ Of these 21 patients, 12 underwent tissue biopsy, and 10 (83%) were confirmed to have extramedullary MS, suggesting potential utility for noninvasive diagnosis. However, larger studies are necessary to validate its clinical relevance.

Currently, MS treatment primarily revolves around AML-based chemotherapy regimens, supplemented by radiotherapy, surgery, immunotherapy, targeted therapy, and HSCT, the latter of which remains the definitive therapeutic approach. Studies indicate that systemic chemotherapy yields longer leukemia-free intervals compared to localized treatment modalities.^[24]^

AML-based chemotherapy, particularly regimens containing cytarabine, is generally the initial treatment of choice, regardless of bone marrow involvement.^[25]^ Allo-HSCT remains the most effective curative approach. Lazzarotto et al^[26]^ reported that 51% of MS patients underwent allo-HSCT, demonstrating significantly improved OS and disease-free survival, especially when performed after achieving complete remission through chemotherapy. Chevallier et al^[27]^ similarly advocated for allo-HSCT, noting that patients who did not receive HSCT typically exhibited OS of <48 months. Furthermore, Taga et al^[28]^ observed that the 4-year survival rate was 100% for patients receiving allo-HSCT post-chemotherapy, compared to 71% for those receiving chemotherapy alone.

Although autologous hematopoietic stem cell transplantation (auto-HSCT) is less commonly employed than allo-HSCT, available data support its efficacy. Simona et al^[29]^ reported that of 11 MS patients undergoing HSCT, 2 of 3 patients receiving auto-HSCT and 4 of 8 patients receiving allo-HSCT survived beyond 5 years. Similarly, Shan et al^[17]^ found that HSCT significantly improved OS compared to chemotherapy alone. In their study, the 2-year OS and progression-free survival for patients receiving auto-HSCT were 78% and 66%, respectively, compared to 65% and 59% for those receiving allo-HSCT. Variations in sample sizes and disease subtypes may account for differences in outcomes, as the auto-HSCT group included more IMS cases.

For recurrent MS, treatment strategies depend on prior therapeutic history. Reinduction chemotherapy remains an option for patients relapsing after chemotherapy, with HSCT consideration upon achieving a second complete remission. For those relapsing post-HSCT, therapeutic options include donor lymphocyte infusion, second transplantation, hypomethylation therapy, and palliative radiotherapy. Recurrent MS presents significant treatment challenges, with generally poor outcomes and reduced survival.

Prognostic studies of MS remain limited due to small sample sizes. Recent large-cohort studies based on public databases have highlighted variations in OS based on lesion site and patient demographics.^[2,30]^ Findings suggest that prognosis worsens with increasing age, and although gender and race have been considered, conclusions remain inconsistent.^[31,32]^ While some studies suggest underlying hematologic diseases may negatively impact prognosis.^[33]^ As can be seen from Table 1, no significant survival differences have been observed between isolated MS and leukemic MS.^[27]^ From Table 2, it can be observed that the survival of IMS is better than that of chronic myelogenous leukemia or myelodysplastic syndromes combined with MS.^[33]^ Overall, timely diagnosis and aggressive treatment remain crucial for improving outcomes in MS patients. Recent development in molecular analysis has provided opportunities for highly targeted therapies. However, the rarity of the disease, and the scarcity of genetic analysis greatly hampered clinical trial development, which means much of the available data relies more on uncontrolled anecdotal reports. Given the rarity of MS, it is essential that further study be through multicenter collaboration. Understanding the genetic profile of MS subtypes will increase the prospect of novel highly targeted therapy. It is also hoped that our expanding knowledge of AML genetics will enhance the understanding of MS mutations and their prognostic significance simultaneously. Prospective multicenter controlled trials are required to further refine management decisions and investigate the role of novel targeted therapies.^[34]^ Due to the rarity of the disease, our research still lacks a large number of specific case support. Therefore, we will continue to follow up on the diagnosis and treatment of MS and strive to provide feasible suggestions for it.

**Table 1:** Prognosis between IMS and leukemic MS.

Outcomes	Overall cohort n = 99	IMS n = 30	Leukemic MS n = 69	*P*
Engraftment	99 (100%)	30 (100%)	69 (100%)	
Time to ANC ≥ 0.5 * 10^9^/L, d (range)		15 (11–30)	18.55 (9–49)	
*Acute GVHD*				
Grade 2–4	23%	27%	35%	
Grade 3–4	9%	3%	11%	
2-year CI of chronic GVHD	45 ± 5%	54 ± 11%	4l ± 7%	.38
5-year OS	48 ± 6%	33 ± 13%	51 ± 7%	.63
	Median: 39 months			
5-year PFS overall	36 ± 5%	30 ± 9%	37 ± 6%	.45
	Median: 17 months			
5-year CI of relapse	40 ± 4%	45 ± 10%	38 ± 6%	.64
5-year CI of NRM	19 ± 4%	17 ± 5%	19 ± 7%	.76

ANC = absolute neutrophil count, GVHD = graft versus-host disease, IMS = isolated myeloid sarcoma, MS = myeloid sarcoma, NRM = nonrelapsemortality, OS = overall survival, PFS = progression-free survival.

**Table 2:** The *P* value of different variables.

Variables	*P* value
Gender	.8756
Location (extremities vs nonextremities)	.4124
Age (≤20 years vs >20 years)	.9197
Chemotherapy vs no chemotherapy	.0009
Radiotherapy vs no radiotherapy	.5760
Surgery vs no surgery	.4475
Combination therapy vs chemotherapy alone	.5652
Chemotherapy with allo-HSCT vs chemotherapy without allo-HSCT	.8647
GS antedating leukemia	.3888
Underlying disease	.0028

Allo-HSCT = allogeneic hematopoietic stem cell transplantation, GS = granulocytic sarcoma, HSCT = hematopoietic stem cell transplantation.

## Author contributions

**Methodology:** Xiaolin Wang.

**Validation:** Xiaolin Wang.

**Writing – original draft:** Xiaolin Wang.

**Writing – review & editing:** Jiaqiang Liu.
